# Oxidative Stress, Kinase Activity and Inflammatory Implications in Right Ventricular Hypertrophy and Heart Failure under Hypobaric Hypoxia

**DOI:** 10.3390/ijms21176421

**Published:** 2020-09-03

**Authors:** Eduardo Pena, Julio Brito, Samia El Alam, Patricia Siques

**Affiliations:** Institute of Health Studies, University Arturo Prat, Avenida Arturo Prat 2120, Iquique 1110939, Chile; samiaelalam@gmail.com (S.E.A.); psiques@tie.cl (P.S.)

**Keywords:** hypobaric hypoxia, cardiac hypertrophy, oxidative stress, kinases, inflammation, heart failure

## Abstract

High altitude (hypobaric hypoxia) triggers several mechanisms to compensate for the decrease in oxygen bioavailability. One of them is pulmonary artery vasoconstriction and its subsequent pulmonary arterial remodeling. These changes can lead to pulmonary hypertension and the development of right ventricular hypertrophy (RVH), right heart failure (RHF) and, ultimately to death. The aim of this review is to describe the most recent molecular pathways involved in the above conditions under this type of hypobaric hypoxia, including oxidative stress, inflammation, protein kinases activation and fibrosis, and the current therapeutic approaches for these conditions. This review also includes the current knowledge of long-term chronic intermittent hypobaric hypoxia. Furthermore, this review highlights the signaling pathways related to oxidative stress (Nox-derived O_2_^.-^ and H_2_O_2_), protein kinase (ERK5, p38α and PKCα) activation, inflammatory molecules (IL-1β, IL-6, TNF-α and NF-kB) and hypoxia condition (HIF-1α). On the other hand, recent therapeutic approaches have focused on abolishing hypoxia-induced RVH and RHF via attenuation of oxidative stress and inflammatory (IL-1β, MCP-1, SDF-1 and CXCR-4) pathways through phytotherapy and pharmacological trials. Nevertheless, further studies are necessary.

## 1. Introduction

Many people are exposed to high altitudes, where more than 140 million of them live permanently at an elevation > 2500 m above sea level, and approximately 40 million individuals are exposed to high altitudes for hours or days [1]. It is important to highlight that high altitude presents an extreme environment (i.e., extreme temperatures, low air humidity and high ultraviolet radiation level), which is challenging for the human body, making it very difficult to adapt to this condition [2,3]. Some ethnic groups, such as the Tibetan people, have undergone adaptive changes in their metabolism [4]. However, the principal impact on humans is due to the low atmospheric pressure and the subsequent proportional decrease in partial oxygen pressure (PO_2_) in the inspired air, generating a reduction in the bioavailability of oxygen in organs, tissues and cells [3]. This condition is termed hypobaric hypoxia.

The classification according to the time of exposure to hypobaric hypoxia is very important, since there are three well-determined conditions: chronic hypobaric exposure that represents people who live permanently at high altitude (Andes and Himalayas) [5] and acute exposure that represents people who are exposed to high altitude for hours or days (tourists and climbers). However, in recent years, a new condition associated with high altitude has been reported in South America as long-term chronic intermittent hypobaric hypoxia; this type of exposure refers to people commuting to work at high altitudes for several days and returning to sea level to rest for the same number of days for years. This condition is termed the “Chilean miner model” of exposure to chronic intermittent hypobaric hypoxia [6].

One of the principal pathologies developed under hypobaric hypoxia, both chronic and intermittent, is high-altitude pulmonary hypertension (HAPH) [7,8]. HAPH is produced initially by hypoxic pulmonary vasoconstriction (HPV), which begins as a compensatory mechanism to distribute the blood to more ventilated areas of the lung [9], mediated through calcium release that acts on Rho-associated protein kinase and the actin-myosin contractile apparatus [10]. Additionally, the activation of molecular pathways and morphological changes, such as uncoupled endothelial nitric oxide synthase (eNOS), pro-inflammatory cytokines such as interleukin 1 and 6 (IL-1 and IL-6), and oxidative stress, among others, lead to pulmonary arterial remodeling and ultimately definitive pulmonary hypertension (PH) [11,12]. Thus, the principal consequence of HAPH is pressure overload (PO)-induced right ventricular hypertrophy (RVH) in the long term [13,14].

Cardiac hypertrophy is defined as an enlargement of the heart wall with an increase in the volume of cardiomyocytes; there are two forms of cardiac hypertrophy, namely, physiological, as occurs in response to exercise, and pathological, as occurs in response to abnormal stress, such as hypertension, pressure overload, endocrine disorders, myocardial infarction, and contractile dysfunction from inherited mutations in sarcomeric or cytoskeletal proteins [15,16]. Initially, this acclimatization response allows normal wall stress function, preserving right ventricle (RV) function, but in the long term, this acclimatization hypertrophy mechanism is overrun; thus, contractile dysfunction and RV dilatation occur, with a subsequent increase in wall stress that stimulates further hypertrophy, leading to a vicious cycle in the impairment of RV performance, which derives in RV failure and eventually leads to death [17,18,19].

Despite the large amount of information that explains RVH by PH-induced PO, we cannot ignore that hypoxia triggers the activation of several molecular pathways that could contribute to both the hypertrophic effect and the dilation process in RV. In fact, studies under hypoxic conditions have determined the important role of oxidative stress [20,21], kinase activation [22,23] and inflammatory processes [24] as possible contributors to RVH development and to the transition to right heart failure (RHF) [25].

However, it is important to highlight that the control mechanisms of RVH and heart failure (HF) in hypobaric hypoxia are still not well understood, and cardiac-directed therapies are rather scarce [23]. Therefore, the aim of this review is to describe and update the knowledge on the molecular pathways involved in RVH and RHF under hypobaric hypoxia, including oxidative stress, protein kinase activation and inflammatory processes, as well as the current therapeutic approaches for these conditions.

## 2. Hypobaric Hypoxia-Induced Oxidative Stress in RVH

In RVH as a consequence of hypobaric hypoxia, several molecular pathways that could be involved in this process are still under study, such as oxidative stress, kinase activation and pro-inflammatory cytokines. In addition, some of these pathways would also be involved in the transition to RHF through apoptosis and fibrosis.

There are oxidative unstable molecules that derive from oxygen, termed reactive oxygen species (ROS). The most well-known ROS molecules are superoxide anion (O_2_·^−^), hydroxyl radical (·OH), peroxyl radicals (ROO·) and alkoxy agents (RO·). Additionally, there are some nonradicals that are either oxidizing or easily converted into oxidative species, including hypochlorous acid (HOCl), ozone (O_3_), singlet oxygen (^1^O_2_), hydrogen peroxide (H_2_O_2_) and peroxynitrite (ONOO^−^) [26].

ROS also play an important role in several cardiac physiological processes, such as the regulation of heart development and cardiomyocyte maturation, cardiac calcium handling [27], as well as excitation and contraction coupling [28,29]. However, when the level of ROS increases due to environmental or chemical stimuli and is not compensated by the endogenous antioxidant system, such as superoxide dismutases (SODs), catalase (CAT), glutathione peroxidase/reductase (GSH-P_X_/R_X_) and peroxiredoxin/thioredoxin [29], oxidative stress is triggered. Additionally, it has been determined that CAT and SODs provide a primary line of defense against ROS generated during this hypoxic condition [30]. Thus, an exacerbated action of ROS not buffered by the antioxidant system could result in damage to DNA, proteins and lipids, as well as activation of the mitochondrial permeability transition pore (MPTP), mitochondrial dysfunction, and cell death [31].

Moreover, studies indicate that oxidative stress and inflammation are closely associated with the progression of myocardial hypertrophy and myocardial infarction [32,33,34]. Studies suggest that oxidative stress can activate nuclear factor-kappa B (NF-kB) in cardiomyocytes, a transcription factor implicated in the regulation of the inflammatory response, which promotes cardiac remodeling and failure [35,36].

Many studies in both humans and animals show an increase in ROS (ROO·, ·OH, O_2_·^−^ and H_2_O_2_) in plasma, lipids (through lipid peroxidation producing malondialdehyde) and tissues after exposure to acute or chronic hypobaric hypoxia (3600–7620 m, 13–8% O_2_) [2,21,37,38,39]. Further support is provided by studies of murine hearts under acute hypobaric hypoxia (10 h, 4570 m, 12% O_2_), showing an upregulation in the transcription levels of pro-oxidant molecules (Ppp1r15b and Xdh) [40]. In other words, oxidative stress under any condition of hypoxia triggers redox signaling pathways in the heart, which in some cases is related to the transcription factor induced by hypoxia, termed hypoxia-inducible factor (HIF)-1α, which will be described in detail later in this review.

One of the principal sources of ROS in the heart is the mitochondria through complex I and complex III [41,42,43]. Mitochondrial-produced oxidative stress in cardiac tissue has been related to the pathology of RVH and HF, since studies have shown that in HF, Ca^2+^ reuptake by the sarcoplasmic reticulum is altered due to dysfunction in sarcoplasmic reticulum Ca^2+^ ATPase 2a (SERCA2a) activity, producing slowed kinetic Ca^2+^ decay in the sarcoplasm, which produces impaired relaxation of the cardiac fiber during diastole. This impairment is compensated by the upregulation of the sarcolemma Na^+^/Ca^2+^ exchanger, resulting in an increased cytosolic Na^+^ concentration in the sarcoplasm [44]. This alteration in ionic homeostasis in the Na^+^ concentration produces alterations in the mitochondria both in energy and ROS production through Na^+^/Ca^2+^, which triggers an energetic deficit and NADPH-induced oxidative stress [45,46]. Moreover, a recent study in mice exposed to intermittent hypobaric hypoxia (5500 m) showed mitochondrial-induced oxidative stress in cardiac tissue [47].

Another important source of ROS in pathological processes is the nicotinamide adenine dinucleotide oxidase (NADPH oxidase) complex, which contains catalytic isoforms called Nox (1–5) and Doux1/2 that form a heterodimer with a lower-molecular-weight subunit called p22phox; this heterodimeric cytochrome is the site of electron transfer to produce O_2_·^−^ or H_2_O_2_ depending on the subunit [33]. Among the Nox isoforms, both Nox2 and Nox4 are the principal isoforms in the heart [33], and their expression are related to the hypertrophy process and HF [20,33,48].

This is supported by studies that show that hypertension-induced hemodynamic stress produces activation of NADPH oxidases, and these products (ROS) can lead to inflammation through NF-kB and activator protein 1 (AP-1) activation, stimulating monocyte chemoattractant protein-1 (MCP-1), also known as CCL2, producing monocyte and macrophage infiltration [49,50].

Hypertension-induced pressure overload in cardiac tissue also induces the recruitment of neutrophil cells, highlighting the fact that the neutrophils are an important cellular source of ROS because they possess significant quantities of NADPH oxidases [51]. In addition, NADPH oxidase-induced H_2_O_2_ and O_2_·^−^ formation has been related to cardiac mechanical stretch through autocrine/paracrine release of angiotensin II (Ang II) [33,52]. On the other hand, it has been demonstrated that Ang II-induced ROS leads to mitochondrial dysfunction and apoptosis of cardiomyocytes [53].

In RVH and RHF, the oxidative stress pathway produce an increase of HIF 1-α activation and mitochondrial complex II-mediated ROS production. On the other hand, the antioxidant enzymes such as SOD and glutathione peroxidase (GP_X_) are not activated under these conditions. However, in left ventricle hypertrophy and failure, there is an increase in NADPH oxidase-mediated ROS production, while antioxidant enzyme activity is increased (SOD, GP_X_) [45]. Therefore, right and left ventricle hypertrophy and failure behave differently.

However, there are controversies regarding cardiac tissue and oxidative stress under hypobaric hypoxia, since it has been shown that intermittent hypobaric hypoxia preconditioning can protect against myocardial ischemia, improve hypoxia resistance and decrease cardiac damage [54]. This is supported by recent studies that present a relationship between iron content, oxidative stress and intermittent hypobaric hypoxia in the cardiac tissue, suggesting that hypoxia exposure may protect tissue by decreasing iron concentration and thereby decrease ROS production through the Haber–Weiss and Fenton reactions, thus preventing cardiac damage [55]. In addition, studies in rats exposed to cycles of hypobaric hypoxia (4600 m) showed a decrease in oxidative stress due to an increase in antioxidant activity mediated by SOD and GSH-Px and improved the contractility of cardiomyocytes, which is associated with an increased ejection fraction [56]. Finally, studies in rats under intermittent hypobaric hypoxia (6000 m) plus exercise showed an improvement in cardiac mitochondrial function and cardiac output and a decrease in brain natriuretic protein (BNP), a cardiac failure biomarker [57]. Despite these controversies, most of the studies concur that excessive ROS induced by hypoxia would be deleterious.

To reconcile this controversy, it must be taken into account that these studies show diverse methodologies and imply a short time and lower degree of hypoxic exposure. In other words, it seems to be a matter of intensity and duration of hypoxia, among other factors. In addition, a systematic review by Coppel et al. [58] of 13 articles compared the normobaric hypoxia and hypobaric hypoxia effects in several aspects, both in the external (time spent in hypoxia, humidity and temperature) and physiological parameters in humans (cardiac output, arterial blood saturation, alveolar ventilation, stroke volume, hematocrit, heart rate and among others), highlighting the fact that alterations in hematocrit, cardiac output, stroke volume, hypoxic cardiac response, hypoxic ventilatory response, lipid peroxidation, and GPx levels showed no differences between both hypoxic conditions. However, this study showed an elevated heart rate and blood pressure in hypobaric hypoxia compared to normobaric hypoxia, while plasma advanced oxidation protein products seem to be major in normobaric hypoxia. Therefore, the standardization of study methods and reporting may aid interpretation of future studies and thereby improve the quality of data in hypoxic conditions [58]; finally, more studies are needed.

## 3. Protein Kinase Activity in RVH under Hypobaric Hypoxia Exposure

Cardiac hypertrophy has also been a focus as a consequence of the role of the superfamily of mitogen-activated protein kinases (MAPKs) as important mediators of hypertrophic stimuli in the heart [22,59]. Canonical MAPK signaling is mediated through three-kinase cascades beginning with an upstream MAPKKK (MEKK) and leading sequentially to MEK and to effector MAPKs, such as c-Jun N-terminal kinases (JNK), p38 and extracellular signal-regulated kinases (ERK) [22]. It is important to highligh that JNK, p38 and ERK are considered redox-sensitive kinases, and their activity is usually reported to regulate inflammation and apoptosis of cardiomyocytes [60,61].

Although studies have shown an important role of p38 and JNK in cardiac hypertrophy [62,63], very few studies have been conducted under hypobaric hypoxia conditions. Some studies regarding the activity of JNK and p38 in rats showed an inactive state of these proteins in the heart, specifically in RVH, under both intermittent hypobaric hypoxia (8 h/d, 5 days, 7000 m) and acute hypobaric hypoxia (9 h, 8000 m) exposure [64,65]. Conversely, p38 presents four isoforms, α, β, γ (also known as Erk6 or SAPK3), and δ (also known as SAPK4), and their expression depends on the tissue and the cardiac process, since only p38α and p38β2 are related to cardiac ventricular hypertrophy [66]. In addition, studies have demonstrated that under another hypoxic condition (chronic normobaric hypoxia), p38, specifically the α subunit, could have a role in heart failure, as will be discussed later.

Likewise, studies in mice with RVH induced by hypobaric hypoxia (5380 m, 10% O_2_) showed a relation with MAP kinase kinase kinase-2 (MEKK2) and the ERK5 pathway, where MEKK2 can coordinately activate signals through MEK5/ERK5 protein kinases, leading to an increase in inflammatory molecules such as interleukin 1β (IL-1β), stromal cell-derived factor 1 (SDF-1), monocyte chemoattractant protein-1 (MCP-1) and C-X-C chemokine receptor type 4 (CXCR-4) triggering the hypertrophic process [23]. In addition, studies have shown an increase in H_2_O_2_-induced mirR-143-3p expression, which increases ERK5 activity in myocardial hypertrophy [67].

However, this is not the only kinase involved in this condition. Hypoxia exposure produces an alteration of protein kinase C (PKC), considered a key regulator in PO-induced cardiac hypertrophy, and its activation can be modulated by ROS derived from mitochondrial complex III [68]. Additionally, it is important to highlight that, similar to p38, PKC has different isoforms (α, β, ε, δ, γ, η, θ and ζ), and their expression depends on factors such as hypoxic stimuli [69], zinc administration [70] and ROS [71].

Consequently, studies in rats with RVH induced by hypobaric hypoxia (380 mmHg, 5500 m) have shown upregulation and activation of PKC-α only in RV [69]. Interestingly, later studies showed that PKC-α produces an increase in galectin-3 expression, which subsequently promotes cardiac fibrosis and HF [72]. Therefore, the results are interesting to determine in future studies the activation and contribution of this PKC-α-galectin-3 pathway in RVH under hypobaric hypoxia conditions.

On the other hand, studies in mouse hearts under hypobaric hypoxia (45 kPa, 11% O_2_) exposure show activation of another PKC isoform (Ca^2+^-independent PKCε), where its activation might have an adaptive and protective role, abolishing mitochondrial impairment in heart tissue [25,73]. Therefore, the assessment of the mechanistic activity and expression of these PKC (PKCε and PKC-α) isoforms in RVH under this particular condition is very challenging for future studies.

It is important to note that the heart comprises a heterogeneous population of cells, including cardiomyocytes, fibroblasts, smooth muscle cells, endothelial cells and immune cells; however, the cross talk between these cells is critical in the initiation, propagation and development of cardiac remodeling leading to HF [74], as will be mentioned below.

## 4. Heart Failure and Inflammation under Hypoxia

In order to achieve a panoramic of the transition to RHF as a consequence of RVH, it must be taken into account that most of the studies about the mechanisms that contribute to this transition come from the left ventricle studies [51] and are not well understood, which is a limitation. Likewise, it must be considered that there are ethnic differences among Tibetan, Andean, and Caucasian populations [75,76].

In the long term, chronic cardiac hypertrophy is sustained and leads to heart failure [77]. Heart failure is defined as an abnormal cardiac structure or function that does not allow the heart to deliver oxygen at a suitable rate for the requirement of the metabolizing tissues [78]. RV initially adapts to the PO produced by pulmonary hypertension by RVH; however, over time, this compensatory mechanism cannot be sustained, and the RV dilates [51]. In addition to PO, there would be other alterations leading to RVH, including other cardiovascular comorbidities, such as myocardiopathies in hypoxia [79]. Cardiac remodeling leads to metabolic alterations and energy supply and deficient energetic supply of ATP [80]. Although a study in diabetic rats under chronic intermittent hypoxia, improve the glucose homeostasis compared to diabetic rats under normoxic condition, through glucose transporter 4 (GLUT 4) translocation [81]. Moreover, in RVH, there is a downregulation of several metabolic pathway regulators critical to ATP production. Thus, there is a change from β oxidation with a downregulation of fatty acid-binding protein, upregulation of AMP kinases and an increased glycogenolysis by upregulation of glycogen synthase kinase-3β and glycogen phosphorylase [45]. All these alterations are linked to a major production of ROS. Additionally, it has been demonstrated that obesity plays a role through respiratory impairment, among others, in the increase of PO and seemingly in RVH and HF [82].

It is important to mention, along with the others factors leading to RHF, metabolic hypoxic myocardiopathy, which is characterized by oxidative damage, cardiac remodeling, ventricular dysfunction [83] and hypertrophy [79]. Notably, this latter disease has also been described in intermittent hypoxia by oxidative damage [79].

Moreover, regarding the relationship of systemic changes by chronic hypoxia with respect to heart failure, there are several issues. At altitude, there are many systemic changes associated with cardiovascular changes involving other systems in charge of cardiovascular functions. Regarding systemic hypertension, its prevalence is controversial because it depends on many factors and confounders, such as ethnicity, altitude and lifestyle [84,85], although the effect of blood pressure itself appears not to be related to right ventricle heart failure, whereas the renin-angiotensin-aldosterone-system (RAAS) does so [45].

In fact, RAAS activation occurs whenever a low output of the left ventricle causes vasoconstriction and increased renal sodium reabsorption and additionally has a profibrotic role in cardiomyocytes [45]. This activation could be explained by chronic right ventricular pressure overload leading to a reduction in the left ventricular stroke volume [86].

Regarding cardiac mechanical loading, a critical and important change is what occurs as erythrocytes increase. The increase in red blood cells up to pathological levels in some individuals, termed chronic mountain sickness, alters the blood viscosity, aggravating pulmonary hypertension and therefore PO, ultimately leading to right ventricle heart failure [76]. Since the right wall tension is a pivotal concept either in RVH or RHF, it could be surmised that this increased PO by hyperviscosity would also affect the right wall tension [87].

Thus, there are some crucial events that characterize this transition from compensatory cardiac hypertrophy to decompensated hypertrophy and HF [88]. The most relevant events in this transition are cardiac fibrosis (through the differentiation of cardiac fibroblasts to myofibroblasts), apoptosis and inflammation [24,25], all of them being associated with oxidative stress.

### 4.1. Fibrosis

Supporting the role of fibrosis, a recent study in rats with RVH induced by hypobaric hypoxia (4500 m) exposure showed a fibrotic process in the RV [89]. A study in mice under other types of hypoxia (chronic normobaric hypoxia, 10% O_2_) showed that animals with PO-induced RVH and HF exhibited increased p38α expression, which was related to several effects, such as increased collagen and smooth muscle actin α (α-SMA) content leading to fibrosis [90]. Additionally, it is important to highlight that p38α is necessary for the differentiation of cardiac fibroblasts to myofibroblasts through the nuclear translocation of myocardin-related transcription factor A (MRTF-A) [90]. However, there are other molecules induced by hypoxia, such as IL-6, endothelin-1 (ET-1) and transforming growth factor α and β (TGF-α and TGF-β), that are related to the transdifferentiation of fibroblasts to myofibroblasts [91], highlighting the key role of IL-6 [92], as will be discussed later.

Myofibroblasts are not part of normal cardiac tissue and appear only following cardiac injury [93]. This type of cell is considered a contractile cell due to the generation of bridges between the internal microfilament and the extracellular fibronectin domains of the myofibroblast through a specialized adhesion complex termed the fibronexus, functioning as a contractile mechanism that produces a force on the surrounding extracellular matrix [93]. Myofibroblasts can also produce and secrete several cytokines, chemokines and growth factors (IL-1α and β, IL-6, IL-10, TNF-α, IL-8, MCP-1, TGF-β, among others) by themselves, which are key in cellular events that lead to inflammatory and fibrotic responses during cardiac stress [74].

It is important to highlight that like fibroblasts, myofibroblasts are not excitable cells; therefore, the increased number of these cells decreases myocyte-to-myocyte coupling through gap junctions [93], which produces a barrier between cardiomyocytes contributing to the development of HF; however, more studies are needed.

Regarding collagen, studies focused on cardiac fibroblasts of RV have shown activation of PKCβ2 and PKCδ, producing an increase in collagen content under hypoxic (10% O_2_) conditions [94].

On the other hand, it has been demonstrated that hypoxia-modulated ORAI1 channels (a nonvoltage-gated channel) that regulate extracellular Ca^2+^ entry through the stromal interacting molecule-1 (STIM1) protein might contribute to RVH and RHF. In addition, these proteins are also expressed in cardiac fibroblasts contributing to cardiac fibrosis [19].

### 4.2. Inflammation

As mentioned above, another important factor is inflammation associated with elevated expression of cytokines, chemokines and transcription factors, such as IL-1β, IL-6, tumor necrosis factor α (TNF-α), MCP-1 and NF-kB, which might lead to right ventricle dysfunction and failure [24] and might also be present in RVH. MCP-1 plays an important role in monocyte recruitment-induced inflammation in cardiac tissue, since studies show that monocyte recruitment is tightly regulated by the interaction between MCP-1 and its receptor CCR2 in myocardial damage [95,96].

Several studies have described that in different PH models, the inflammatory molecule IL-6 is increased both in plasma and RV tissue, which could be involved in cardiac function and morphology [36]. This is supported by a study by Nehra et al. [14] in rats with RVH induced by hypobaric hypoxia (7620 m) showing an increase in circulating levels of TNF-α and IL-6, and these molecules were associated with a decompensated transition to RHF. Moreover, a recent study in mice with transverse aortic constriction shows new insight into hypoxia-induced mitogenic factor (HIMF), a cytokine-like protein that could induce cardiac hypertrophy, fibrosis and myofibroblast differentiation through IL-6 [97], which results in an interesting pathway to be analyzed in the transition to HF under this particular condition. However, a study in 15 healthy humans (age: 39 ± 10.2; 4 women, 11 men; BIM 23.6 ± 2) of European ancestry exposed for 72 h at 3830 m (acute hypobaric hypoxia) determined no changes in the plasma levels of IL-1β, IL-6 and NF-kB. However, the ROS production rate (O_2_·^−^ and ·OH) and markers of ROS damage, such as lipid peroxidation, 8-isoPGF2α, 8-hydroxy-2′-deoxyguanosine (products of DNA oxidation) and protein oxidative carbonylation, were significantly increased over time [21].

### 4.3. Apoptosis

Regarding apoptotic events, studies have shown that hypobaric hypoxia exposure produces an increase in cardiac apoptosis in both rat and mouse models [14,98]. In addition, studies have shown that oxidative stress may further accelerate the development of RHF through the induction of more cardiac apoptosis [51]. In addition, cytokines, such as TNF-α, are also involved in the apoptotic process in cardiomyocytes [49]. It is important to highlight that this molecule is increased by ROS [99]; therefore, we can hypothesize that hypoxia-induced ROS could trigger cardiac apoptosis through TNF-α. This is supported by recent studies in mice and rats that demonstrated that the administration of ROS scavenger molecules, such as nitronyl nitroxide radicals, curcumin and nanocurcumin, under hypobaric hypoxia conditions, prevents oxidative stress and attenuates cardiac apoptosis [14,98].

### 4.4. HIF-1α

Likewise, sustained PO leads to p53 accumulation, triggering the apoptosis process and inhibiting antiapoptotic HIF-1 activity [77]. HIF-1 is one of the main regulators to consider in any hypoxic response [100] and is able to mediate the transcription of over 200 target genes [77]. In general, HIF-1 plays a major role in immune functions, oxidative stress, metabolic reprogramming, angiogenesis and antiapoptosis processes [77]. Moreover, studies have shown that the transition to maladaptative pulmonary artery hypertension-induced RVH is associated with a decrease in HIF-1α through mitochondrial-derived ROS, thus suppressing angiogenesis [101].

Under normoxic conditions, the HIF-1α subunit is hydroxylated at two proline residues, mediated by the enzyme prolyl hydroxylase (PHD) [102]. The Von Hippel–Lindau (VHL) ubiquitin ligase complex recognizes prolyl-hydroxylated HIF-α subunits and marks them for proteasomal degradation. However, under hypoxic conditions, PHD hydroxylation activity decreases, producing HIF-1α stabilization [100].

It is important to note that several cytokines and inflammatory processes, such as inflammatory cell migration (monocytes and macrophages) and monocyte differentiation to macrophages, are modulated by HIF-1α under hypoxic conditions [77,103]. Moreover, monocyte maturation to macrophages is accompanied by the production of cytokines and growth factors [96] since macrophages contribute to the release of different cytokines, such as TNF-α, IL-1β, IL-6, IL-12 and IL-23 [24]. In addition, the cytokine TNF-α produced by macrophages stabilizes and increases the activity of HIF-1α, generating a positive feedback that leads to further differentiation of monocytes to macrophages [103]. It is important to mention that NF-kB is a key mediator in TNF-α-induced HIF-1α stabilization and activation [103]; hence, the relation between hypoxia-induced factors, such as HIF-1α, and inflammatory pathways in macrophages are potentiated.

However, there are controversies regarding the monocyte/macrophage (MΦ) effects in the cardiac remodeling process and HIF-1α under hypoxic conditions, since a study showed that HIF-1α-induced MΦ accumulation suppresses fibrosis in the heart through oncostatin-m (OSM), a pleitropic cytokine member of the IL-6 family [104].

On the other hand, several studies have shown that MΦ-produced OSM plays an important role in several inflammatory processes, including cardiomyocyte remodeling, fibrotic diseases and dilated cardiomyopathy [105,106]. Moreover, it is important to highlight that OSM displays the broadest signaling that involves several protein kinases activated by hypoxia, including ERK, p38, JNK, and PKC-δ [105], which has been described in this review. Nevertheless, more investigation will help reconcile this controversial statement.

**Figure 1:**
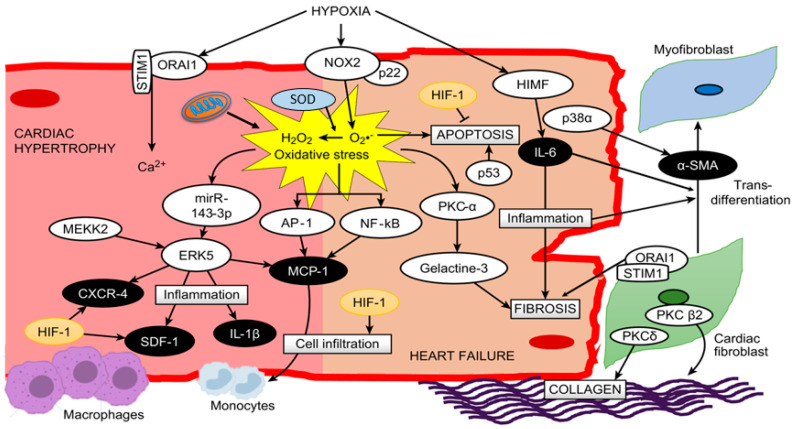
A proposed scheme summarizing all described molecular pathways and cells involved in cardiac hypertrophy and heart failure under hypoxia.

As mentioned before, both chronic and intermittent hypoxia induce several HIF-1α target genes and can upregulate proteins such as inducible nitric oxide synthase (iNOS), ET-1 and angiotensin-converting enzyme (ACE) [107]. iNOS seems to have a protective role in the heart against oxidative stress and apoptosis [108], while both ET-1 and ACE are related to cardiac hypertrophic effects [51,109]. Nevertheless, there is a lack of studies that show a relationship between these molecules (ACE, ET-1 and iNOS) and RVH under hypobaric hypoxia conditions, although studies of another type of hypoxia demonstrated that the use of angiotensin II receptor blockers produced a reduction in normobaric hypoxia-induced RVH [110]. However, the role of HIF-1 in cardiac hypertrophy and HF remains to be determined [77]. A proposed schematic in cardiac myocytes summarizing all these molecular pathways is depicted in Figure 1.

## 5. Treatment Approaches

### 5.1. Phytotherapy

Based on the important role of oxidative stress, fibrosis and inflammatory processes, current studies have focused on new therapies to limit ROS production and enhance ROS detoxification as means of ameliorating cardiac disease outcomes [29]. This can be supported by studies in rats exposed to acute hypobaric hypoxia (8000 m), which showed that the administration of an ancestral medicine of Tibet (*Rhodiola crenulata*) for preventing high-altitude illness decreases arginase 1 activity, oxidative stress and apoptosis and increases eNOS activity in heart tissue [65].

Studies by Nehra et al. [14] have demonstrated that nanocurcumin protects the heart against hypobaric hypoxia effects even better than curcumin. Additionally, the combination of preconditioning physical training plus oral administration of antioxidant nanocurcumin formulation decreases the RVH, circulatory markers of cardiac damage and oxidative stress in rats exposed to hypobaric hypoxia (7620 m, 8% O_2_) even more than each treatment alone [111].

The administration of *Hippophae rhamnoides* L. extract reduced oxidative stress (malondialdehyde level), fibrosis and NF-kB expression in the hearts of rats exposed to hypobaric hypoxia [112].

### 5.2. Pharmacological and Gene Therapy

Studies in transgenic mice with hypobaric hypoxia-induced RVH showed that target disruption of MEKK2, through insertion of the neomycin resistance gene in the coding sequence, attenuates ERK5 abundance and activation, which is consistent with an attenuation of RVH [23]. It is important to highlight that in this study, a reduction in inflammatory molecules related to the cardiac hypertrophy process, such as IL-1β, MCP-1, SDF-1 and CXCR-4, was also observed [23]. Therefore, this study presents the potential benefit of therapies targeting MEEK2 related to RVH and HF in this particular condition. Moreover, studies show the importance of MCP-1 both in cardiac remodeling and failure, since it is produced by several molecular pathways induced by hypoxia, as has been described before, where hypoxia-induced oxidative stress appears to play a relevant role in the increase in MCP-1 in the cardiac tissue. Therefore, it is noteworthy to propose MCP-1 as a future molecular target in the treatment of hypoxia-induced RVH and RHF.

Another interesting approach to improve cardiac outcomes is the use of hemoglobin-based oxygen carriers to deliver carbon monoxide in mice exposed to intermittent hypobaric hypoxia (8 h/day, 7 d; 5500 m), which showed improved cardiac function, reduced myocardial apoptosis, and attenuated mitochondrial oxidative stress under treatment, highlighting an anti-inflammatory effect through a decrease in IL-6 and TNF-α levels in plasma [47].

A study in rats exposed to hypobaric hypoxia (5500 m, 380 mm Hg) with administration of salubrinal, a specific inhibitor of eIF2α phosphatase enzyme, showed that this molecule ameliorates RVH and cardiac fibrosis and attenuates oxidative stress and ET-1 levels in serum [113]. All these treatments mentioned would contribute to further support of the pivotal role of ROS, TNF-α and IL-6 molecules in the transition from compensatory cardiac hypertrophy to decompensated hypertrophy and HF under hypobaric hypoxia conditions.

On the other hand, a recent study in rats exposed to hypobaric hypoxia (10% O_2_) administered soluble macromolecules termed drag-reducing polymers and determined that these drugs attenuate right ventricular dysfunction and hypertrophy [114]. Another drug used to decrease PH-induced RVH in mice under hypobaric hypoxia (0.5 atm) is bosentan, an endothelin receptor antagonist; however, bosentan also induced a decrease in macrophages, IL-6, and MCP-1 in the lung [115]. Finally, we might consider bosentan as a new therapeutic agent to ameliorate cardiac remodeling and HF because of the decrease in the same molecules described both in RVH and HF induced by hypoxia exposure.

Most interesting treatments are focused on the RV under normobaric hypoxia. A study in rats with Sugen/hypoxia (10% O_2_) condition-induced PH showed that the use of dual-blocked endothelin A and B receptor (ET_a_ and ET_B_) treatment (macitentan) improved cardiac output and function, decreased cardiac hypertrophy, and increased perfusion and microvessels in the RV [116]. In addition, a recent study determined improved cardiac performance through administration of a specific ET_B_ blocker (BQ-788) in mice under extreme normobaric hypoxia (5–15% O_2_) [117].

**Figure 2:**
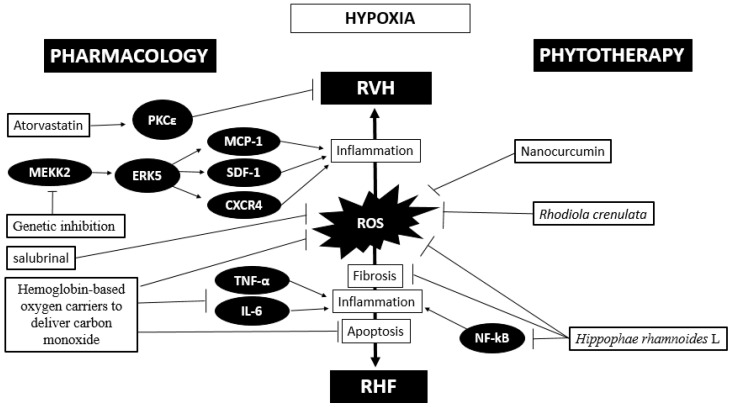
Phytotherapy and pharmacological approaches described in right ventricular hypertrophy (RVH) and heart failure (HF) under hypoxia; the arrow represent an inductor factor and T arrow correspond to an inhibitory factor.

A recent study showed that the administration of atorvastatin increases PKCε, increasing its cardioprotective effects through the inhibition of miR-31 under intermittent hypoxia (5% O_2_) conditions [118]. This therapy opens a promising avenue to attempt the search for molecules to block deleterious pathways or induce protective pathways. Finally, these pharmacological treatments, either alone or combined, under hypoxic conditions seem to be very challenging. A proposed scheme of the phytotherapy and pharmacological approaches is depicted in Figure 2.

In summary, oxidative stress plays a pivotal role in promoting the activation of some kinases in inducing an inflammatory status in hypoxia-induced RVH and HF. Pharmacological therapy, phytotherapy and gene therapy have been attempted with promising results that need to be validated.

## Abbreviations

PO_2_	Partial Oxygen Pressure
HAPH	High-Altitude Pulmonary Hypertension
HPV	Hypoxic Pulmonary Vasoconstriction
eNOS	Endothelial Nitric Oxide Synthase
IL-1	Interleukin 1
IL-6	Interleukin 6
PH	Pulmonary Hypertension
PO	Pressure Overload
RVH	Right Ventricular Hypertrophy
RV	Right Ventricle
RHF	Right Heart Failure
HF	Heart Failure
ROS	Reactive Oxygen Species
O_2_·^−^	Superoxide Anion
·OH	Hydroxyl Radical
ROO·	Peroxyl Radicals
RO·	Alkoxy Agents
HOCl	Hypochlorous Acid
O_3_	Ozone
^1^O_2_	Singlet Oxygen
H_2_O_2_	Hydrogen Peroxide
ONOO^−^	Peroxynitrite
SODs	Superoxide Dismutases
CAT	Catalase
GSH-P_X_/R_X_	Glutathione Peroxidase/Reductase
MPTP	Mitochondrial Permeability Transition Pore
NF-kB	Nuclear Factor-Kappa B
HIF-1α	Hypoxia-Inducible Factor-1α
Ca^2+^	Calcium
SERCA2a	Sarcoplasmic Reticulum Ca^2+^ ATPase
Na^+^	Sodium
NADPH	Nicotinamide Adenine Dinucleotide
AP-1	Activator Protein 1
MCP-1	Monocyte Chemoattractant Protein-1
Ang II	Angiotensin II
BNP	Brain Natriuretic Protein
MAPKs	Mitogen-Activated Protein Kinases
JNK	C-Jun N-Terminal Kinases
ERK	Extracellular Signal-Regulated Kinases
IL-1β	Interleukin 1β
SDF-1	Stromal Cell-Derived Factor 1
CXCR-4	C-X-C Chemokine Receptor Type 4
PKC	Protein Kinase C
A-SMA	Smooth Muscle Actin A
MRTF-A	Myocardin-Related Transcription Factor A
ET-1	Endothelin-1
TGF-α	Transforming Growth Factor α
TGF-β	Transforming Growth Factor β
STIM1	Stromal Interacting Molecule-1
TNF-A	Tumor Necrosis Factor A
HIMF	Hypoxia-Induced Mitogenic Factor
PHD	Prolyl Hydroxylase
VHL	Von Hippel-Lindau
MΦ	Monocyte/Macrophage
OSM	Oncostatin-M
iNOS	Inducible Nitric Oxide Synthase
ACE	Angiotensin-Converting Enzyme
ET_a_	Endothelin A Receptor
ET_b_	Endothelin B Receptor
RAAS	Renin-Angiotensin-Aldosterone-System
GLUT4	Glucose Transporter 4
GPx	Glutathione peroxidase
